# The impact of central foveal thickness and integrity of the outer retinal layers in the visual outcome of uveitic macular edema

**DOI:** 10.1186/s40942-021-00306-8

**Published:** 2021-04-27

**Authors:** Carlos Alvarez-Guzman, Andres Bustamante-Arias, Maria F. Colorado-Zavala, Alejandro Rodriguez-Garcia

**Affiliations:** 1 Tecnologico de Monterrey, School of Medicine and Health Sciences, Institute of Ophthalmology and Visual Sciences, Ocular Immunology & Uveitis Service, Monterrey, Mexico; 2 Hospital Zambrano Hellion, TecSalud, Av. Batallon de San Patricio No. 112. Col. Real de San Agustin, San Pedro Garza Garcia, N.L. C.P. 66278 Mexico

**Keywords:** Uveitis, Macular edema, Cystoid macular edema, Diffuse macular edema, Subretinal fluid, SD-OCT, Central foveal thickness, EPIS, interdigitation zone, Visual loss

## Abstract

**Background:**

To analyze the relationship between the central foveal thickness (CFT) and the integrity of the ellipsoid portion of inner segments (EPIS) and interdigitating zone (IZ) retinal layers in the visual outcome of uveitic macular edema (UME).

**Methods:**

Prospective, observational, and cross-sectional study of eyes with UME. Spectral-domain optical coherence tomography (SD-OCT) macular morphological pattern, CFT, and integrity of the outer retinal layers were analyzed. We arranged the data by EPIS or IZ integrity and contrasted it with student t-test (quantitative variables) and Fisher exact test or χ² distribution (categorical variables) to evaluate visual impairment and retinal measures. Receiver operator curve (ROC) estimation and logistic regression (probit) assessed if the sample´s variance could be associated with IZ or EPIS integrity.

**Results:**

We included 145 SD-OCT macular scans from 45 patients at different stages of UME. Cystoid macular edema (CME) increased the risk of severe (P ≤ 0.0162) and moderate visual loss (P ≤ 0.0032). The highest CFT values occurred in patients with moderate (478.11 ± 167.62 μm) and severe (449.4 ± 224.86 μm) visual loss. Of all morphological patterns of macular edema, only CME showed a statistically significant relationship with severe visual impairment (44.92%, p = 0.0035, OR 4.29 [1.62–11.4]). Likewise, an increased probability of severe visual loss correlated negatively with both, IZ (37.93%, P ≤ 0.001, OR 10.02) and EPIS (38.98%, P ≤ 0.001, OR 13.1) disruption. A CFT > 337 μm showed a higher probability of IZ (AUROC = 0.7341, SEN 77.59%, ESP 65.52) and EPIS (AUROC = 0.7489, SEN 76.37%, ESP 65.12%) loss of integrity. Moreover, when BCVA reached 0.44 LogMAR (≤ 20/50 Snellen eq.), it was more likely to have IZ (AUROC = 0.8706, ESP 88.51%, SEN 77.59%) and EPIS (AUROC = 0.8898, ESP 88.3%, SEN 76.27) disruption.

**Conclusions:**

Significantly increased CFT has a higher probability for EPIS and IZ disruption, which significantly increases the risk for irreversible visual loss in eyes with UME. Evaluating these layers’ integrity by optical coherence tomography helps predict the visual outcome and make the right therapeutic decisions.

*Trial registration* The study was registered on April 13, 2020, at the Instituto Tecnologico y de Estudios Superiores de Monterrey Research Committee (License No. COFEPRIS 20 CI 19 039 002), project registration No. P000338-CAVICaREMU-CI-CR002, and the Ethics Committee (License No. CONBIOETICA 19 CEI 011-2016-10-17), project registration No. P000338-CAVICaREMU-CEIC-CR002

## Background

Uveitis encompasses a diverse group of ocular inflammatory disorders, leading to potential sight-threatening complications such as cataracts, glaucoma, and macular edema, the latter being the most common cause of visual impairment in up to 41% of patients [1].

Uveitic macular edema (UME) is more commonly found in chronic disorders where vitreous inflammation occurs, particularly in intermediate uveitis (25–70%), panuveitis (35%), and posterior uveitis (20%) [2]. The anatomical subtypes of macular edema include diffuse macular edema (DME), cystoid macular edema (CME), serous retinal detachment (SRD) or subretinal fluid (SRF), and vitreoretinal interface abnormalities [3–5]. Although epiretinal membrane (ERM) and vitreomacular traction (VMT) are not caused by fluid accumulation, their contribution to the pathogenesis of macular thickening has granted their consideration as part of the UME clinical spectrum by some authors [6]. According to the vast majority of studies, CME is the most frequent type causing both legal blindness (29%) and visual impairment (41%) [1]. On the other hand, the increase in retinal thickness affects visual acuity and is considered an important contributor to UME´s visual loss [4, 6, 7]. However, there is a need for more objective ways to correlate the degree of visual loss with the macular microstructural changes to make the right therapeutic decisions and better judge these patients’ visual prognosis. Macular spectral-domain ocular coherence tomography (SD-OCT) provides qualitative and quantitative data to confirm, classify the morphology, measure the thickness, and monitor the treatment response [3, 7]. One of many advantages of SD-OCT is that it provides rapid acquisition of noninvasive high-resolution images of all retinal layers. Four hyperreflective lines are identified in the outer retina by SD-OCT, the external limiting membrane (ELM), the inner and outer photoreceptor segments junction (IS/OS), also known as the ellipsoid portion of inner segments (EPIS/Ellipsoid zone), the cone outer segment tips (COST) or interdigitating zone (IZ), and the retinal pigment epithelium (RPE) [2]. The morphologic analysis of the integrity or disruption of these retinal layers as clinical markers for predicting visual outcome has been the subject of previous investigations [4, 6, 7]. The disruption of the IS/OS junction, ELM, and IZ have a deleterious effect on visual function [8–11].

This study aimed to determine the correlation between visual acuity with the morphologic type of macular edema, the increase in central foveal thickness (CFT), and the integrity or disruption of the outer retinal layers (EPIS and IZ) in eyes at different stages of UME from different etiologies. Also, to analyze how these macular SD-OCT quantitative and morphologic parameters may serve as visual loss predictors.

## Methods

We designed a prospective, observational, and cross-sectional study in patients diagnosed with uveitis of different etiologies seen at our clinic. Informed consent was obtained from every patient in whom an SD-OCT macular analysis was required to analyze/confirm the presence of macular edema or monitor its therapeutic response/resolution. The study was approved by the Ethics and Research Committees of our institution following the Declaration of Helsinki’s tenets. Inclusion criteria comprised patients ≥ 18 years with macular edema secondary to diverse uveitis forms who voluntarily agreed to participate in the study. Exclusion criteria consisted of patients unwilling to participate in the study, eyes with end-stage maculopathy including atrophic scarring, choroidal neovascularization, the concurrence of other causes of macular edema like diabetic retinopathy, previous retinal vascular occlusive disease, and poor-quality SD-OCT scans (weak signal SSI ≤ 45). Patients with any comorbidity affecting vision, such as amblyopia, irregular astigmatism, corneal opacity, significant cataract (≥ 2 + LOCS III grade), posterior capsule opacification, vitreous haze, optic nerve atrophy, advanced glaucoma, and macular hole, or scarring.

Routine assessment for all eyes included standardized Snellen best-corrected visual acuity (BCVA), tonometry, biomicroscopy, and indirect fundus examination. Macular SD-OCT (RTVue-100, Optovue® Inc., Fremont, CA. USA) analysis was performed by the same examiner (SIS). MM5 macular maps were performed to every eye for quantitative CFT measurements. This map covers a 5 × 5 mm-grid area; in the central 3-mm area, the scans are spaced 0.25 mm apart, and in the 3-mm to 5-mm-region, the scans are spaced 0.5 mm apart. The Crossline SD-OCT scan maps were obtained for morphologic features (edema subtype and outer retinal layers integrity).

According to the inflammation site, uveitis was classified following the Standardization of Uveitis Nomenclature (SUN) criteria [12]. The study eyes were divided according to loss of vision or not, where “visual loss” was defined as a BCVA ≤ 20/30. This cut-off value was set to 20/30 to increase the correlation sensitivity of EPIS and/or IZ outer retinal layer disruption. Furthermore, the degree of visual impairment was categorized into four groups: no visual loss (20/20–20/25), mild (20/30–20/50), moderate (20/60−20/100), and severe visual loss (≥ 20/200). The Snellen BCVA fraction was converted to the logarithm of minimal-angle resolution (logMAR) units for the statistical analyses.

Macular edema was defined as a central foveal thickness (CFT) > 260 μm in the MM5 scan map, supported by indirect ophthalmoscopy showing absence of the foveal reflex, macular elevation, and cysts formation or by a late macular hyperfluorescence, or dye pooling with a “petaloid pattern” during fluorescein angiography. The morphologic patterns of UME include diffuse and cystoid macular edema and subretinal fluid accumulation, as described by previous studies [4, 7]. DME was defined as a spongy appearance accompanied by small low-reflective areas of the retinal layers with increased macular thickness; CME was clearly defined as low-reflective intraretinal spaces (cysts) separated by thin high-reflective septa. SRF consists of the neuro-sensorial retina separation from the retinal pigment epithelium (RPE), seen as a well-defined low-reflective space [2]. The SD-OCT crossline scan maps were used to evaluate the macular edema subtypes and the integrity or disruption of the outer retinal layers. These retinal layers, which are vulnerable to different macular disorders, may serve as hallmarks for the photoreceptors´ integrity. The EPIS and IZ appear as two parallel hyperreflective lines localized between the external limiting membrane and the RPE. Disruption was defined as a discontinuity of the hyperreflective bands corresponding to EPIS or IZ layers within the crossline scan map of 500 microns. RPE atrophy was also analyzed in all scans and was considered whenever there was a loss of the correspondent hyperreflective band, or a choroidal hyper transmission phenomenon was observed.

SD-OCT macular scans were taken at different stages of UME. For example, one patient contributed with several SD-OCT scans taken during the uveitis clinical course. So, we could perceive the behavior of UME’s quantitative and morphologic features and their impact on visual acuity outcome during CFT and outer retinal layers integrity changes.

The data were captured in Excel spreadsheets (Windows® version-2017, Microsoft Corp.) and analyzed using R-Statistics version 3.6.2 for Windows (GNU-Free Software Foundation. Boston, MA. USA). Descriptive statistics for quantitative variables were calculated as a central tendency (average), dispersion (standard deviation), and frequencies for categorical variables. To evaluate the main objectives of the study, we arranged the data by IZ or EPIS integrity. We contrasted them with the student t-test (quantitative variables) and Fisher exact test, or χ² distribution (categorical variables) for visual impairment and retinal measures. Furthermore, we performed a receiver operator curve (ROC) estimation and logistic regression (probit) to evaluate if the sample’s variance could be associated with IZ or EPIS integrity.

## Results

The study evaluated 145 SD-OCT macular scans from 45 patients with UME. Of these, 28 (62.22%) were women, and 17 (37.78%) men with a mean age of 42.61 (SD ± 17.66) years. Regarding uveitis features, 70.34% of the patients had bilateral uveitis. Anterior (35.17%) and intermediate (28.28%) uveitis were the most common forms, with pars planitis being the most frequent cause (Table 1).

**Table 1:** Clinical features and etiology of eyes with uveitic macular edema

Classification criteria of uveitis	No. eyes (n = 145)	Percentage
Anatomic classification		
Anterior	51	35.17
Intermediate	41	28.28
Panuveitis	28	19.31
Posterior	25	17.24
Laterality		
Unilateral	43	29.66
Bilateral	102	70.34
Ocular inflammatory activity		
Active	68	46.90
Controlled	77	53.10
Etiology		
Pars planitis	41	28.28
Vogt-Koyanagi-Harada disease	27	18.62
Idiopathic anterior uveitis	18	12.41
HLA-B27-associated anterior uveitis	17	11.72
Retinal Vasculitis	15	10.34
Multifocal choroiditis	6	4.14
Juvenile idiopathic arthritis-associated anterior uveitis	5	3.45
Others^a^	16	11.04

^a^Others: acute zonal occult outer retinopathy, idiopathic chronic anterior uveitis, granulomatosis with polyangiitis, punctate inner choroidopathy

**Fig. 1:**
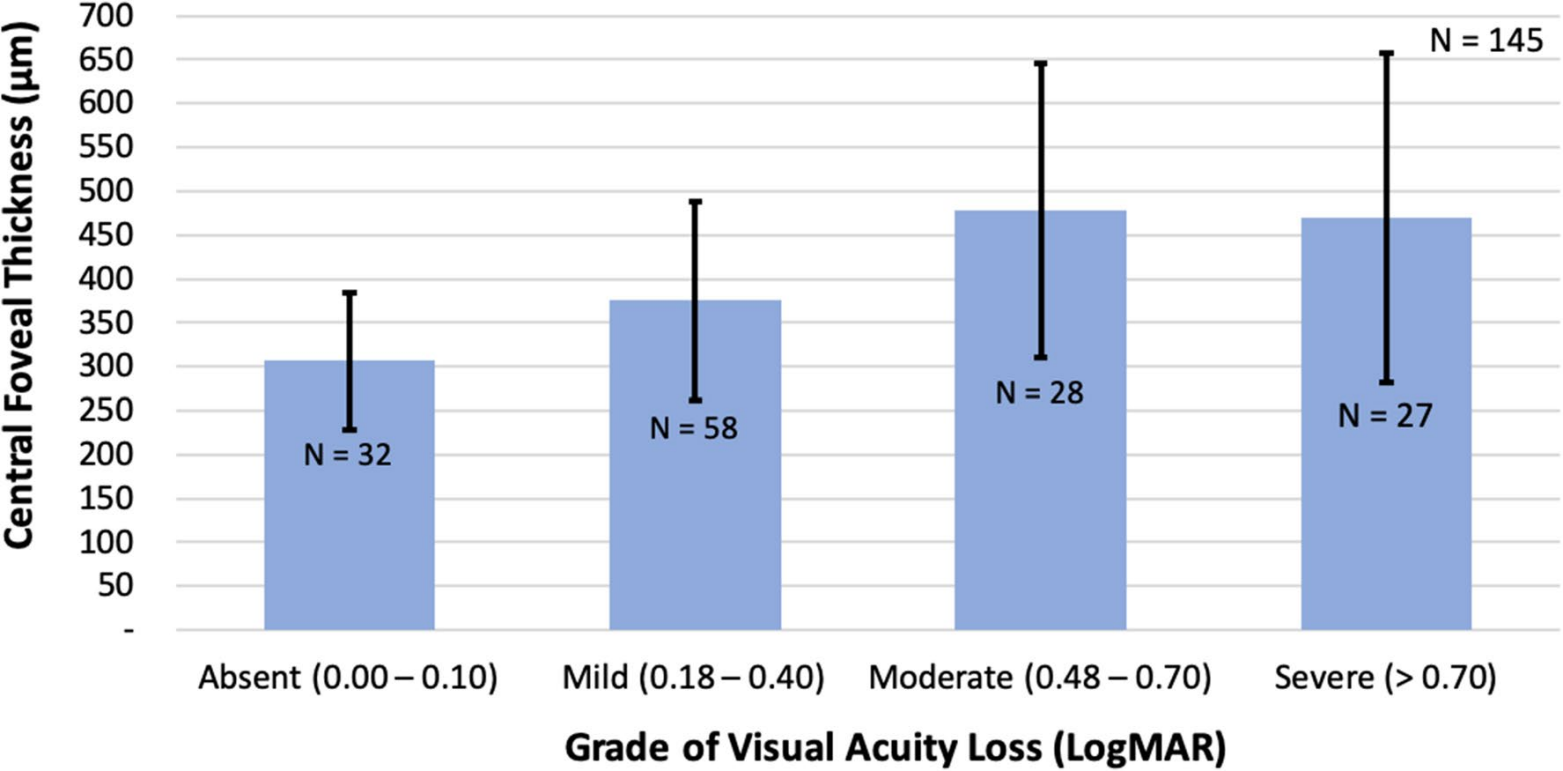
Relationship between central foveal thickness with the grade of visual loss in uveitic macular edema

The prevailing morphologic macular edema patterns were CME (51.03%), DME (40.70%), and SRF (8.52%). No eyes with ERM were included in the study (Table 2). The mean CFT and BCVA for each morphologic subtype of UME analyzed are also shown in Table 2. The CME group showed the lowest BCVA (0.63 ± 0.41 LogMAR, 20/80 Snellen eq.) with a mean CFT of 460.32 ± 143.96mm. Up to 77.93% of eyes experienced visual loss, considering a BCVA ≥ 0.18 LogMAR (≤ 20/30 Snellen eq.) as the threshold. Concerning the degree of visual loss, a total of 32 (22.07%) eyes showed no visual loss (LogMAR range, 0.00–0.10), while the remaining 113 (77.93%) eyes had some degree of visual loss (LogMAR range, ≥ 0.18): 40.0% of eyes had a mild visual loss, 19.31% moderate, and 18.62% severe visual loss. The mean CFT of all eyes was 397.53 ± 149.35 μm. The highest CFT values were observed in patients with moderate (478.11 ± 167.62 μm) and severe (469.85 ± 187.43 μm) visual loss, while eyes with no visual loss displayed a lower CFT (306.81 ± 78.04 μm) (Fig. 1).

**Table 2:** Distribution of macular edema subtype and outer retinal layer status according to the anatomical site of inflammation and their relationship with central foveal thickness and best-corrected visual acuity

Type of uevitis	CME (N = 74)	DME (N = 59)	SRF (N = 12)	Status of EPIS		Status of IZ	
				Integrity (N = 86)	Disruption (N = 59)	Integrity (N = 87)	Disruption (N = 58)
Anterior	28 (37.84%)	23 (38.98%)	0 (0.0%)	28 (32.56%)	23 (38.98%)	27 (31.03%)	24 (41.38%)
Intermediate	22 (29.73%)	19 (32.20%)	0 (0.0%)	20 (23.26%)	21 (35.59%)	20 (22.99%)	21 (36.20%)
Posterior	15 (20.27%)	7 (11.86%)	3 (25.0%)	21 (24.42%)	4 (6.78%)	21 (24.14%)	4 (6.90%)
Panuveitis	9 (12.16%)	10 (16.96%)	9 (75.0%)	17 (19.76%)	11 (18.64%)	19 (21.84%)	9 (15.52%)
Mean CFT (mm)	460.3 ± 143.9	296.7 ± 61.4	505.8 ± 201.9	349.7 ± 125.3	467.2 ± 155.1^a^	352.5 ± 127.7	465 ± 155.0^a^
Mean BCVA (Snellen eq.)	0.63 ± 0.41 (20/80)	0.24 ± 0.33 (20/30)	0.52 ± 0.39 (20/60)	0.24 ± 0.26 (20/30)	0.77 ± 0.40^a^ (20/125)	0.26 ± 0.28^a^ (20/30)	0.76 ± 0.41^a^ (20/125)

*CME *cystoid macular edema, *DME *diffuse macular edema, *SRF *subretinal fluid accumulation, *EPIS *ellipsoid portion of inner segments, *IZ *interdigitating zone, *CFT *central foveal thickness, *BCVA *best-corrected visual acuity

^a^Statistically significant (p < 0.001, student-t test)

The hypothesis tests showed a statistically significant relationship between CME (44.92%, p = 0.0035, OR 4.29 [1.62–11.4]) and severe visual loss, while the other macular edema patterns did not show an increased risk of significant visual impairment.

Likewise, an increased probability of severe visual loss correlated significantly with both, EPIS (38.98%, P ≤ 0.001, OR 13.1) and IZ (37.93%, P ≤ 0.001, OR 10.02) disruption. Also, a higher probability of moderate visual loss correlated significantly with both, EPIS (37.29%, P ≤ 0.001, OR 7.93) and IZ (39.66%, P ≤ 0.001, OR 10.78) disruption.

Regarding the morphologic status of the outer retinal layers in UME, the overall integrity of the photoreceptor layers was found to be similar for both EPIS (59.30%) and IZ (60.00%), and these figures did not differ significantly between all the different subtypes of macular edema (Table 2). The mean CFT was significantly higher in eyes with EPIS (467.2 ± 155.1mm) and IZ (465 ± 155.0mm) disruption compared to their respective intact layer counterparts (p < 0.001). Also, the mean BCVA was significantly lower in eyes with EPIS (0.77 ± 0.4) and IZ (0.76 ± 0.41) disruption compared to their intact layer counterpart (p < 0.001) (Table 2). Moreover, a total of 135 (93.10%) scans did not show any RPE atrophic changes, including 86 scans with no outer retinal layer disruption. On the other hand, of the 59 eyes with EPIS and/or IZ disruption, only 10 (16.94%) of them with CME showed RPE atrophy. The mean CFT of these eyes was 537.4 mm, and the BCVA was 0.92.

**Fig. 2:**
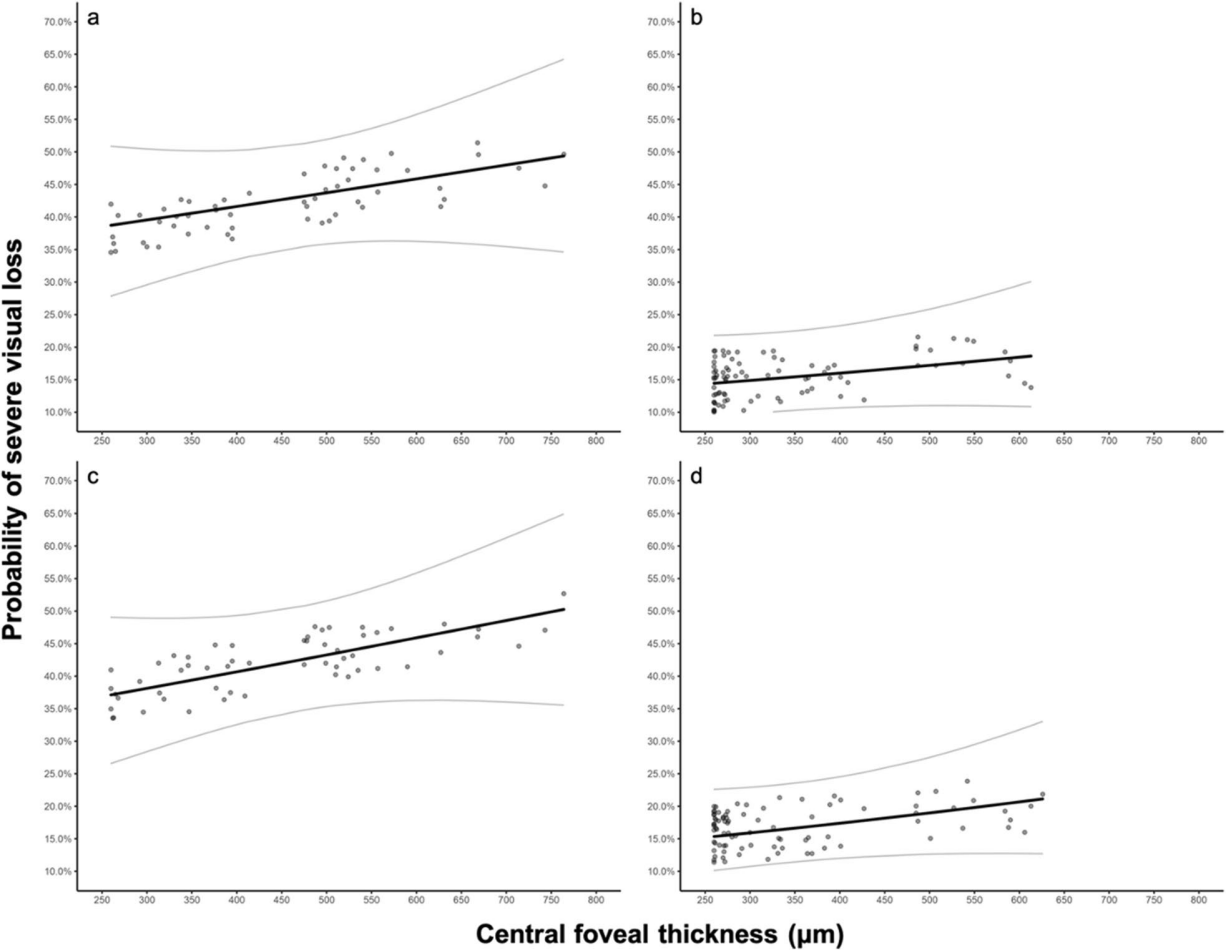
Probability of severe visual loss as a function of the CFT and the integrity or disruption of EPIS and IZ outer retinal layers. The logistic regression model predicts increased risk and a higher probability of severe visual impairment (3.74 OR with CI 95% = 2.07–6.77) (P = 0.001) when there is EPIS disruption (**a**), and a low risk of severe visual loss when this retinal layer is preserved (**b**). In the same manner, the logistic regression model predicts an increased risk (3.26 OR with CI 95% = 1.85–5.74) (P = 0.001) of visual impairment if IZ gets disrupted (**c**), and a low risk of severe visual loss when this retinal layer is preserved (**d**). *EPIS *ellipsoid portion of inner segments, *IZ *interdigitating zone

Macular thickness above 317 μm (AUROC = 0.663, SEN 81.5%, SPE 46.6%) increased the risk of severe visual impairment. Further analyses of macular thickening by logistic regression showed that as the retinal thickness increases, so does the visual deterioration risk. EPIS (OR 3.74, CI 95% = 2.07–6.77 (P ≤ 0.001) or IZ layer (OR 3.26, CI 95% = 1.85–5.74) (P ≤ 0.001) disruption increased the visual loss probability when the variables were added to the logistic regression model (Fig. 2).

## Discussion

Macular edema is a common visually-impairment complication of different forms of uveitis, including anterior uveitis associated with the HLA-B27 haplotype, juvenile idiopathic arthritis, pars planitis, retinal vasculitis, Behcet's disease, among many others [13, 14].

SD-OCT imaging permits a rapid, noncontact meticulous analysis of the macular microstructural changes observed in patients with macular edema. It provides accurate quantitative foveal thickness measurements and detailed morphologic characteristics of the edema and all retinal layers at the macula.

**Fig. 3:**
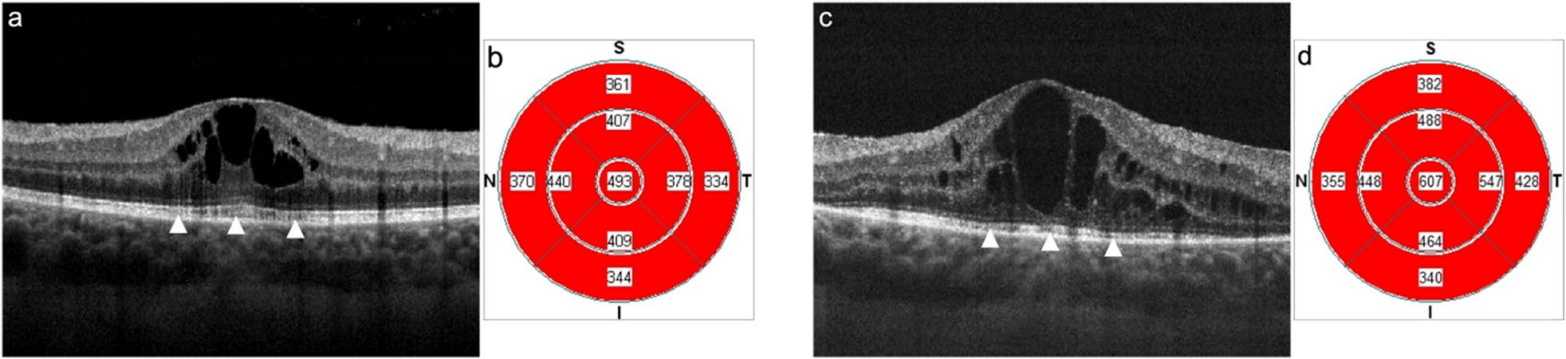
Initial macular SD-OCT (**a**) of a 43-year-old female diagnosed with idiopathic retinal vasculitis, revealing CME with a CFT of 493 μm (**b**) and a BCVA of 20/20. After four years of combined systemic immunosuppressive/biologic therapy and three sustained-release 700mm dexamethasone phosphate intravitreal implantations, CME (**c**) with a CFT of 607 μm (**d**) and a BCVA of 20/30 persist. The white arrowheads identify the integrity of EPIS and IZ retinal layers in both crossline SD-OCT images. *SD-OCT *spectral-domain optical coherence tomography, *CME *cystoid macular edema, *CFT *central foveal thickness, *BCVA *best corrected visual acuity, *EPIS *ellipsoid portion of inner segments, *IZ *interdigitating zone

**Fig. 4:**
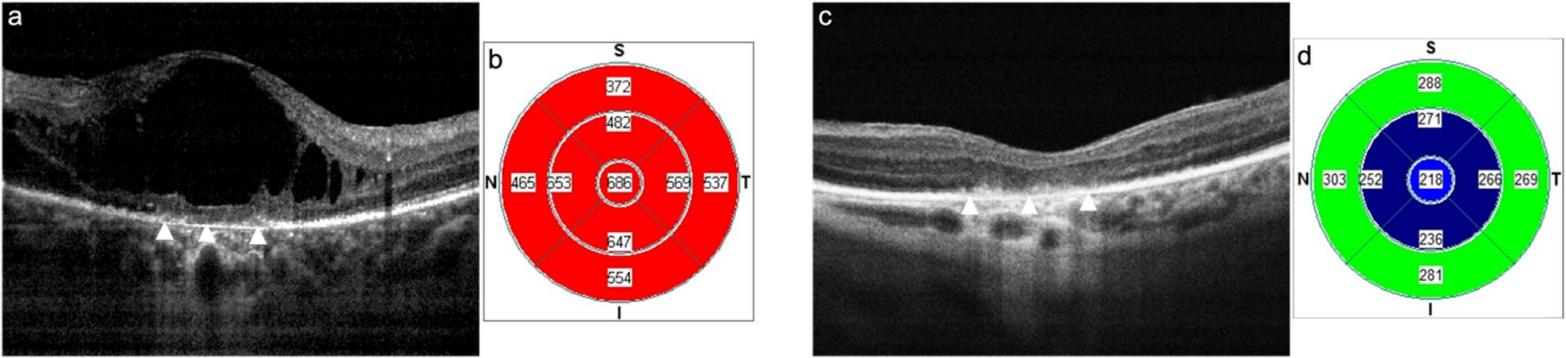
Initial macular SD-OCT (**a**) of a 68-year-old female diagnosed with idiopathic chronic anterior uveitis, revealing CME with a CFT of 686 μm (**b**) and a BCVA of 20/200. After four years of systemic immunosuppressive therapy, periocular and intravitreal corticosteroid injections, there is complete resolution of the CME (**c**) with a CFT of 218 μm (**d**), but with persistent visual loss (BVCA = 20/100). The white arrowheads identify the disruption of both EPIS and IZ retinal layers in the initial and final crossline SD-OCT images, along with subfoveal retinal pigment epithelium (RPE) atrophy in the final image. *SD-OCT *spectral-domain optical coherence tomography, *CME *cystoid macular edema, *CFT *central foveal thickness, *BCVA *best corrected visual acuity, *EPIS *ellipsoid portion of inner segments, *IZ *interdigitating zone

The primary motivation to perform the present study was to try to elucidate the clinical finding of reasonable preserved BCVA in the presence of significant UME characterized by intact EPIS and IZ layers by SD-OCT (Fig. 3). In contrast, other chronic uveitic eyes show poor or no visual recovery after adequate treatment solving the macular edema (regaining a normal CFT) but with EPIS and/or IZ disruption (Fig. 4). Therefore, we decided to analyze by SD-OCT the correlation between BCVA with the morphologic subtypes of edema, the increase in CFT, and the integrity or disruption of the outer retinal layers (EPIS and IZ) in eyes with UME from different etiologies and at different stages of the disease.

The present study population was predominantly composed of women with bilateral intraocular inflammation (Table 1). Our population’s demographic pattern was similar to that previously reported [5, 14, 15].

According to similar studies, we have found a significant correlation between visual loss with CME, the most frequent morphologic type of UME [2, 3, 5, 14, 16–18]. Furthermore, in the present study, CME increased the risk of severe visual impairment by 4-fold (44.92%, p = 0.0035, OR 4.29 [1.62–11.4]), while the other patterns of macular edema did not increase the risk of visual loss. Previous studies agree with our results concerning a lack of correlation between DME and poor BCVA or visual loss [3, 5, 7]. While most studies have found that SRF does not correlate with visual loss or uveitis severity [5–7, 19, 20], others found increased visual loss in eyes with SRF combined with CME or DME [3, 4, 21]. We found SRF in insufficient numbers (8.52%) to throw statistical conclusions regarding its influence on visual loss and recovery.

Regarding the impact that increased macular thickness has on the visual acuity of eyes with UME, we found similar results to previous reports, which showed that the higher the CFT, the worse BCVA [5, 18, 19]. More interestingly, we noticed that the higher the CFT, the better chance to have EPIS and IZ disruption (Table 2). In this respect, eyes with a CFT > 337mm showed significantly high sensitivity and specificity for layer disruption with a 74.8% EPIS and 73.4% IZ accuracy. The higher probability of outer retinal layer disruption after significant CFT increase has not been reported in the literature. Additionally, an increased risk of moderate and severe visual loss also correlated with EPIS and IZ disruption. Further analyses of macular thickening by logistic regression showed that as the retinal thickness increases, so does the visual deterioration risk. Therefore, differing from previous reports analyzing which parameter, CFT or outer retinal layers integrity, serves as a better prognostic factor for visual outcome, we found that a significantly increased CFT has a higher probability for EPIS and/or IZ disruption, and this, in turn, significantly increased the risk for visual loss in eyes with UME.

Interestingly, an En Face SD-OCT study investigating the structure-function relationship to predict UME’s visual outcome found a strong correlation between preserved retinal tissue on baseline OCT and visual outcome after inflammatory CME resolution. This finding implies that the preserved retinal tissue at baseline is a better predictor than macular thickness or volume for future visual potential after CME resolves following therapy. A hypothesis formulated to explain the lack of visual recovery after macular edema resolves suggests that extracellular fluid accumulation within the retina may disrupt neuronal and synaptic function. Hence, significant macular edema with a high CFT value could produce permanent macular tissue damage, including significant axon elongation and disruption of the outer retinal layers, resulting in irreversible visual loss [22].

Several studies first showed that the percentage of EPIS disruption in patients with diabetic macular edema and those with epiretinal membrane formation related to uveitis has a strong correlation with visual loss, concluding that the alteration in the integrity of this layer is a predictive factor for a poor visual outcome [23–25]. Later on, a negative correlation between EPIS (IS/OS) disruption with visual loss was first described in patients with UME, concluding that this outer retinal layer’s integrity appeared to be essential for the functional evaluation [5]. On the other hand, a previous SD-OCT analysis performed in eyes with inflammatory macular edema found a correlation between irregularity or loss of IZ (then known as the third highly reflective band by OCT) and poor visual acuity [18]. Additionally, a stronger association of IZ disruption over EPIS with visual loss was also reported in vitrectomized eyes from different etiologies, including uveitis for epiretinal membrane delamination [9].

Regarding the integrity of both EPIS and IZ layers, there is only one study consisting of 52 eyes with UME that found a correlation of EPIS and IZ disruption with poor vision, besides an increase in CFT and/or the presence of CME [6]. This study highlights the IZ integrity as the most critical factor for UME´s visual prognosis [6]. In the present study, no significant differences were found in terms of the correlation between BCVA and EPIS or IZ integrity to determine which one is a better predictor for visual loss. Moreover, our findings insinuate that BCVA correlates negatively with the disruption of both EPIS and IZ outer retinal layers in eyes with UME. The disruption of EPIS or IZ increased the visual loss probability when the variables were added to the logistic regression model, suggesting that we may encounter severe visual impairment at lesser retinal thickness when the aforementioned retinal conditions are present (Fig. 2).

Concerning the potential influence of RPE atrophic changes seen in some eyes with chronic UME on the visual outcome; a careful examination of our cases with no visual recovery despite anatomic resolution of edema revealed that only in 10 scans (16.94%), loss of outer retinal layer integrity was associated with RPE atrophy (Fig. 4.). Furthermore, although the mean CFT of these eyes reached 537.4 mm and visual acuity was significantly affected, this infrequent finding on eyes with EPIS/OZ disruption suggests that the RPE atrophy associated with prominent and prolonged CME did not play a determinant role in the final visual outcome on most of these eyes.

## Conclusions

In conclusion, few studies have evaluated the relationship between the CFT and the integrity of the outer retinal layers in UME eyes and their association with visual loss. Recognition of such association permits more objective outcome predictors to take a right therapeutic decision and monitor macular edema’s evolution in uveitis patients. Therefore, in the routine clinical care of these patients, we regularly perform SD-OCT, and in some cases, fluorescein angiography on them to evaluate both the activity of the disease and the development of posterior segment complications. This study agrees with previous findings that an increased CFT and the CME subtype are associated with visual loss in uveitis patients. More importantly, a higher foveal thickness was associated with EPIS and IZ disruption, which in turn, may produce a significant visual loss.

One limitation of this study was that only eyes with UME detected by SD-OCT were considered for analysis; it would be desirable to study eyes with subclinical UME detected earlier by fluorescein angiography in a longitudinal way to analyze CFT changes and their relationship with alterations in the outer retinal layers integrity and visual outcome.

We have found that CME and substantially increased CFT have a higher probability of EPIS and IZ disruption. However, it may be possible that the rate of EPIS/IZ disruption could be overestimated in eyes with a higher CFT due to OCT transmission artifacts produced by the presence of significant edema that makes EPIS and IZ less identifiable. Nevertheless, such SD-OCT quantitative and morphologic alterations significantly affect the patients’ visual outcome.

The importance of conducting a systematic SD-OCT morphologic analysis of UME arises from identifying eyes that fail to improve visual acuity after multiple intravitreal, periocular, or sustained-release corticosteroid injections based only on the observation of frank macular cysts or increased CFT. Under such circumstances, if EPIS or IZ are disrupted, despite a significant reduction of CFT after anti-inflammatory therapy, it is unlikely that visual acuity will improve if we only pursue the reduction of macular thickness. On the other hand, in eyes with increased CFT but with intact photoreceptor layers, anti-inflammatory treatment would be desirable to improve vision and avoid further visual impairment.

## Data Availability

The datasets generated and analyzed during the current study are not publicly available because they contain personal information of patients kept under The Privacy Protection of Data in the hands of a Third-Party National Law (Mexico), but are available from the corresponding author on reasonable request.

## Abbreviations

UME: Uveitic macular edema
DME: Diffuse macular edema
CME: Cystoid macular edema
SRD: Serous retinal detachment
SRF: Subretinal fluid
ERM: Epiretinal membrane
VMT: Vitreomacular traction
SD-OCT: Spectral-domain optical coherence tomography
ELM: External limiting membrane
IS/OS: Inner and outer photoreceptor junction
EPIS: Ellipsoid portion of inner segments
COST: Cone outer segment tips
IZ: Interdigitating zone
RPE: Retinal pigment epithelium
BCVA: Best-corrected visual acuity
CFT: Central foveal thickness
MM5: Macular map 5 × 5mm
OR: Odds ratio
AUROC: Area under the receiver operating characteristics
